# The PLOS ONE collection on machine learning in health and biomedicine: Towards open code and open data

**DOI:** 10.1371/journal.pone.0210232

**Published:** 2019-01-15

**Authors:** Leo A. Celi, Luca Citi, Marzyeh Ghassemi, Tom J. Pollard

**Affiliations:** 1 MIT Laboratory for Computational Physiology, Massachusetts Institute of Technology, Cambridge, United States of America; 2 School of Computer Science and Electronic Engineering, University of Essex, Colchester, United Kingdom; 3 University of Toronto, Computer Science and Medicine, Toronto, Canada; 4 Vector Institute, Toronto, Canada; PLOS, UNITED KINGDOM

## Abstract

Recent years have seen a surge of studies in machine learning in health and biomedicine, driven by digitalization of healthcare environments and increasingly accessible computer systems for conducting analyses. Many of us believe that these developments will lead to significant improvements in patient care. Like many academic disciplines, however, progress is hampered by lack of code and data sharing. In bringing together this PLOS ONE collection on machine learning in health and biomedicine, we sought to focus on the importance of reproducibility, making it a requirement, as far as possible, for authors to share data and code alongside their papers.

## Introduction

In its strategic plan for data science released in June of this year, the National Institute of Health stated that we “stand at a unique moment of opportunity in biomedical research” [1]. "Advances in [data] storage, communications, and processing" offer an opportunity for technologies such as machine learning to yield "transformative changes for biomedical research over the coming decade". This promise, alongside more accessible data and open-source software, has created a surge of studies in machine learning in health and biomedicine. Our collection provides a glimpse of things to come.

At the time of authoring the introduction to the collection, we had received over 100 submissions, of which we selected a subset across topics including primary care, acute care, medical imaging, and global health. Papers already accepted cover a diverse range of topics, including areas such as early detection of glaucoma, prediction of survival using health records, localization of ossification in radiographs, and risk stratification in neuroblastoma using transcriptomics data [2–5]. In all cases, the authors have included a link to their code and instructions for accessing data. Many more papers are under review.

While we have no doubt that applications of machine learning in health and biomedicine will have a tremendous impact on the outcomes of many future patients, we share concerns that the rigor of research is often tainted by the environment that drives it [6,7]. To establish trust in research findings, greater transparency of data, protocols, and code is desirable [8,9]. In the Call for Papers for this collection, we emphasised that "Data underlying the study’s findings will be a requirement of publication, per the PLOS data policy" and that authors would be "responsible for providing, upon submission, the source code needed to replicate their findings" [10].

Journals such as *PLOS ONE* have had firmly worded policies on the importance of data and code sharing for many years, so these requirements are nothing new. Nevertheless, our experience has shown that policies on data and code sharing are often weakly enforced. It is rare for a published study in any journal to be associated with publicly available code and data, and it is even rarer for the code and data to be sufficiently well-curated to enable a third party to reproduce the study. Statements such as “code and data are available from the authors upon request” are the norm, rather than the exception. Such statements are typically misleading [11,12].

We therefore present this collection with an emphasis on reproducibility, to set a precedent for publishers and researchers alike on how open publishing policies can be applied to studies in health and biomedicine. With support from *PLOS ONE* staff, we have worked hard to ensure that all of the papers in the collection are associated with public code. While some data could not be shared publicly, all papers should at the very least provide institutional contact information for data requests in line with PLOS ONE's data availability policy. Despite best efforts, not all of the code and data is as cleanly organized or presented as we would like, but we hope that readers will respond positively to the good intentions of all authors and help data and code sharing to become common practice.

## Reproducibility

Computational reproducibility is the ability to repeat an analysis of a given data set and obtain sufficiently similar results ([13–16] and references therein). It requires having the complete software environment available, properly documented full source code, and the original data. Ideally the reader should be able to inspect, modify and apply the code under modified parameter settings to reproduce the results and explore the robustness of the algorithm to the values of its parameters [17]. In recent years, platforms designed for the development of software such as GitHub, GitLab, and BitBucket, have been adopted by the scientific community as ways to distribute the code that accompanies scientific papers. Initially little more than web-based front ends for source control systems such as “git”, they evolved into integrated solutions that can render markdown documents and Jupyter notebooks, which can be used to visually present the results together with the code used to obtain them [18]. A number of platforms have been created to facilitate computational reproducibility of code shared through such platforms. One such example is Code Ocean, which allows readers to directly interact with code by running it within a widget embedded within an article [19]. Another example is Binder, which enables an investigator to quickly reproduce a computational environment using data and code shared online [20,21].

A potentially groundbreaking algorithm and its code implementation only really benefit the community and the wider society if they can be applied to new data and adapted to similar problems. Therefore reproducibility should be taken a step further by aiming for reusability [22], which enables the application of the methodology to new questions or new data so that future studies build upon past studies and science progresses faster. Reusability requires that the authors make the additional step of explaining and documenting how some decisions were taken or how some parameters were chosen based on the data. The authors should make the extra effort to make the code easy to maintain and to extend. As any derived code should be similarly distributed, the issue arises of a proliferation of different versions of similar code. This is where the use of repository management services, like GitLab for example, can make a difference by allowing researchers to clone existing code, modify it to suit their needs and possibly integrate potential improvements back into the original repository through a pull request. These services allow the community to track the evolution of the initial piece of code accompanying a paper into a widely used toolbox through collaborative science.

## Data sharing

Data sharing in medical research is advocated for reasons including verification of results and for unlocking the opportunity to address complex medical questions through the creation of large multi-center datasets [23–26]. In the U.S., the National Institutes of Health (NIH) has required data sharing for all large funding grants since 2003 while the National Science Foundation (NSF) has required research grant proposals to include data sharing plans since 2011. Similar policies have been introduced by the UK research councils [27] and by the European Commission [28]. Piwowar (2011) examined studies funded by the NIH and the NSF and found that data sharing remains infrequent in spite of the recommendations by the funding agencies [29]. Even in the field such as genomics with mature policies, repositories and standards, research data sharing levels are low and increasing only slowly.

In a recent survey of patients participating in clinical trials, a large majority (82%) indicated that they perceived the benefits of sharing deidentified data to outweigh the negatives [30]. 93% of respondents in the survey indicated willingness to allow their clinical trial data to be shared with scientists. A dominant theme in responses to the survey was the need for clinical data to help others as much as possible. Many of the respondents urged greater cooperation and less competition among scientists. The feeling that overt competitive behaviour can hamper research progress has been reflected in scientist sentiment: one study found that researchers who perceive their fields as particularly competitive are more likely to withhold data [31]. Numerous studies have suggested that data withholding can have a detrimental effect on scientific training and research [30–34].

Over the last decade, a significant proportion of journals have adopted guidelines for authors that explicitly require data associated with studies to be shared. Simple statements of willingness to share data by investigators rarely translate to true availability when data is requested by independent scientists [9], which motivates a need for sharing by more formal methods (for example, sharing via public repositories). Even where data is findable and accessible, maximum value can be gained where it is interoperable (for example, using standardised vocabularies) and reusable (for example, well described with an open license), as outlined in the FAIR Principles [35].

In a 2011 survey of papers published in 50 popular research journals, less than 10% of investigators made their raw data available [32]. Investigators typically cite concerns around patient privacy as the primary reason for withholding data. Privacy is a serious matter and it is appropriate that this concern curtails data sharing to an extent, but approaches that help to address privacy concerns while allowing data to be shared are emerging. These approaches typically include combinations of deidentification (removal of information that allows data to be attributed to a patient), statistical methods such as differential privacy to obscure details, and access control through protected networks.

The development of clear and effective policies to regulate data sharing is an ongoing task for governing organisations. Notably, the European Union (EU) General Data Protection Regulation (GDPR) went into effect in May 2018, with the goal of harmonizing data protection across the EU. It applies to data pertaining to any EU resident, regardless of where that data was processed. The policy was crafted with the understanding that health data should be a public good, but the penalty for breach of patient privacy is so steep (up to 4% of annual global revenue or 20 million Euros) that there are concerns that it will curtail momentum around data sharing [36]. The GDPR does not apply to anonymous or anonymized data, but it allows for significant room in the interpretation of key aspects of data protection, including when data are considered anonymized; what safeguards are sufficient for processing data under the research exemption; and what further limitations should be set on processing personal data for research purposes [37].

Despite the obstacles to data sharing, authors of studies submitted to the collection found solutions. In reviewing papers in their approaches to data sharing, we applied some general principles: open is better than closed; some data must be restricted, but this should not prevent it from being citable and discoverable; where data is restricted, synthetic or sample data should be provided; it is better to share data in specialist archives than as supplementary files. In general, authors of papers submitted to the collection were receptive to our data sharing requests. Several submissions that simply stated that data would be “available upon request” in initial versions, for example, were subsequently updated to provide links to datasets in public repositories. In one study, the authors determined that while the raw dataset could not be shared for privacy reasons, it was possible to provide code to enable its reproduction. In many cases, simple rewording of data availability statements helped to clarify how a researcher might obtain a protected dataset from a host institution.

## Code sharing

There are specific cases where there is potential for private information to leak into code—for example, caution might be needed in sharing a word embedding generated from detailed patient notes—but in general code does not suffer the privacy risks associated with data. For this reason, one might expect code to be widely shared. In practice we find this is not the case, in short because effort outweighs reward. Familiar excuses for not sharing code include: people might find bugs; the code isn't clean enough, and; supporting users will be too much work [38,39]. For most studies there is a nugget of truth in each of these points. As a community we should strive to share anyway, accepting that bugs will be found; code can always be improved; and expectations must be managed. There are many excellent reasons to share code and countless ways to do so [40,41].

We believe that reviewing well-documented code can provide as much insight into a study as reviewing a paper itself. Code that is not clear and well-managed raises questions about the quality of a study, even if the paper itself is well-written and apparently scientifically sound. The PLOS ONE editorial team assessed the availability of code for every paper considered for inclusion in the collection. Similar to our approach for data, several basic principles were applied: code should be open unless there are exceptional circumstances; protection of intellectual property will not be cause for exception; user guidelines and a license are required; a fixed version of the code that underpins the study should be permanently archived and linked from the paper with a persistent identifier such as a Digital Object Identifier (DOI).

When code accompanies a paper submitted to a journal, how rigorously should it be peer reviewed? Ideally, the code should be reviewed with the same rigour as the paper, but relying on an already-stretched pool of referees to do this work is a large ask. For this collection, code was made available to referees during the review process where possible, but there was no expectation for it to be reviewed and we did not attempt to execute the code. By ensuring that code is available and discoverable from articles, however, we create the opportunity for post-publication review.

## Closing remarks

The papers in this collection present a range of machine learning applications in health and biomedicine. To our belief, these papers go beyond what is typical in this field in terms of data and code sharing. For the research community, we hope that the collection sets a standard that encourages sharing more widely. For journal editors, we intend to demonstrate that authors are generally open to sharing when prompted. Finally, for organizations looking to fund machine learning applications in healthcare, we urge investments into the development of tools and platforms that promote reproducibility.

## References

[1] National Institutes of Health (NIH). NIH releases strategic plan for data science. Retrieved November 1, 2018, from https://www.nih.gov/news-events/news-releases/nih-releases-strategic-plan-data-science.

[2] Kucur ŞS, Holló G, Sznitman R (2018) A deep learning approach to automatic detection of early glaucoma from visual fields. PLOS ONE 13(11): e0206081 10.1371/journal.pone.0206081 30485270 PMC6261540

[3] Steele AJ, Denaxas SC, Shah AD, Hemingway H, Luscombe NM (2018) Machine learning models in electronic health records can outperform conventional survival models for predicting patient mortality in coronary artery disease. PLOS ONE 13(8): e0202344 10.1371/journal.pone.0202344 30169498 PMC6118376

[4] Koitka S, Demircioglu A, Kim MS, Friedrich CM, Nensa F (2018) Ossification area localization in pediatric hand radiographs using deep neural networks for object detection. PLOS ONE 13(11): e0207496 10.1371/journal.pone.0207496 30444906 PMC6239319

[5] Maggio V, Chierici M, Jurman G, Furlanello C (2018) Distillation of the clinical algorithm improves prognosis by multi-task deep learning in high-risk Neuroblastoma. PLOS ONE 13(12): e0208924 10.1371/journal.pone.0208924 30532223 PMC6285384

[6] Economist The. (2013, 10). Unreliable research: Trouble at the lab. The Economist. Retrieved from http://www.economist.com/node/21588057/print

[7] Ioannidis J. P., Greenland S., Hlatky M. A., Khoury M. J., Macleod M. R., Moher D., et al (2014). Increasing value and reducing waste in research design, conduct, and analysis. Lancet, 383(9912), 166–75. 10.1016/S0140-6736(13)62227-8 24411645 PMC4697939

[8] Ioannidis J. P. (2018) All science should inform policy and regulation. PLoS Med. 2018;15(5):e1002576 Published 2018 May 3. 10.1371/journal.pmed.1002576 29723196 PMC5933781

[9] Baker M. (2016). Why scientists must share their research code. Nature. 10.1038/nature.2016.20504

[10] PLOS. Call for Papers (2018). Retrieved November 1, 2018, from https://blogs.plos.org/speakingofmedicine/2018/03/09/call-for-papers-machine-learning-in-health-and-biomedicine/

[11] Savage CJ, Vickers AJ (2009) Empirical study of data sharing by authors publishing in PLOS journals. PLOS One 4: e7078 10.1371/journal.pone.0007078 19763261 PMC2739314

[12] Stodden V., Seiler J., and Ma Z. PNAS 3 13, 2018 115 (11) 2584–2589; 10.1073/pnas.1708290115 PMC5856507 29531050

[13] Stodden V. and Miguez S. (2014) ‘Best Practices for Computational Science: Software Infrastructure and Environments for Reproducible and Extensible Research’, Journal of Open Research Software, 2(1), p. e21 10.5334/jors.ay

[14] Piccolo S. R., & Frampton M. B. (2016). Tools and techniques for computational reproducibility. Gigascience, 5(1), 30 10.1186/s13742-016-0135-4 27401684 PMC4940747

[15] Oudeyer P.-Y., & Merrick K. (eds) (2016). CDS Newsletter—The of the Technical Committee on Cognitive and Developmental Systems. Volume 13, number 2, Fall 2016.

[16] Alnoamany Y., & Borghi J. A. (2018). Towards computational reproducibility: researcher perspectives on the use and sharing of software (No. e26727v1). PeerJ Preprints. 10.7717/peerj-cs.163 PMC7924683 33816816

[17] Buckheit J. B., & Donoho D. L. (1995). Wavelab and reproducible research In Wavelets and statistics (pp. 55–81). Springer, New York, NY 10.1007/978-1-4612-2544-7_5

[18] Torvalds L., & Hamano J. (2010). Git: Fast version control system. Retrieved November 1, 2018, from http://git-scm.com.

[19] Code Ocean. Retrieved November 1, 2018, from https://codeocean.com/.

[20] Binder. Retrieved November 1, 2018, from https://mybinder.org/

[21] The eLife blog: Introducing Binder 2.0. Retrieved November 1, 2018, from https://elifesciences.org/labs/8653a61d/introducing-binder-2-0-share-your-interactive-research-environment

[22] Varoquaux G. (2016). Beyond computational reproducibility, let us aim for reusability. Newsletter of the IEEE Technical Committee on Cognitive and Developmental Systems, 13(2).

[23] Auffray C., Balling R., Barroso I., Bencze L., Benson M., Bergeron J., et al (2016). Making sense of big data in health research: towards an EU action plan. Genome medicine, 8(1), 71 10.1186/s13073-016-0323-y 27338147 PMC4919856

[24] Kohane, I. S., Van Wingerde, F. J., Fackler, J. C., Cimino, C., Kilbridge, P., Murphy, S., et al. (1996). Sharing electronic medical records across multiple heterogeneous and competing institutions. In Proceedings of the AMIA Annual Fall Symposium (p. 608). American Medical Informatics Association. PMC2233175 8947738

[25] Dye C., Bartolomeos K., Moorthy V., & Kieny M. P. (2016). Data sharing in public health emergencies: a call to researchers. Bull World Health Organ, 94(3), 158 10.2471/BLT.16.170860 26966322 PMC4773943

[26] Margolis R., Derr L., Dunn M., Huerta M., Larkin J., Sheehan J., et al (2014). The National Institutes of Health's Big Data to Knowledge (BD2K) initiative: capitalizing on biomedical big data. Journal of the American Medical Informatics Association, 21(6), 957–958. 10.1136/amiajnl-2014-002974 25008006 PMC4215061

[27] UKRI. Common principles on data policy. Retrieved November 1, 2018, from https://www.ukri.org/funding/information-for-award-holders/data-policy/common-principles-on-data-policy/

[28] European Commission. Research & Innovation—Participant Portal H2020 Online Manual. Retrieved November 1, 2018, from http://ec.europa.eu/research/participants/docs/h2020-funding-guide/cross-cutting-issues/open-access-data-management/data-management_en.htm

[29] Piwowar H (2011) Who shares? Who doesn’t? Factors associated with openly archiving raw research data. PLoS One 6: e18657 10.1371/journal.pone.0018657 21765886 PMC3135593

[30] Mello MM, Van Lieou BS, Goodman SN (2018) Clinical trials participants’ views on the risks and benefits of data sharing. N Eng J Med 378:2202–2211. 10.1056/NEJMsa1713258 PMC6057615 29874542

[31] Vogeli C, Yucel R, Bendavid E, Jones L, Anderson M, et al (2006) Data withholding and the next generation of scientists: results of a national survey. Acad Med 81: 128–136. 16436573 10.1097/00001888-200602000-00007

[32] Alsheikh-Ali AA, Qureshi W, Al-Mallah MH, Ioannidis J (2011) Public availability of published research data in high-impact journals. PLoS One 6: e24357 10.1371/journal.pone.0024357 21915316 PMC3168487

[33] Campbell EG, Clarridge BR, Gokhale M, Birenbaum L, Hilgartner S, et al (2002) Data withholding in academic genetics: evidence from a national survey. Jama 287: 473–480. 11798369 10.1001/jama.287.4.473

[34] Blumenthal D, Campbell EG, Gokhale M, Yucel R, Clarridge B, et al (2006) Data withholding in genetics and the other life sciences: prevalences and predictors. Acad Med 81: 137–145. https://www.ncbi.nlm.nih.gov/pubmed/16436574 16436574 10.1097/00001888-200602000-00006

[35] Wilkinson Mark D., Dumontier Michel, Aalbersberg IJsbrand Jan, Appleton Gabrielle, Axton Myles, et al "The FAIR Guiding Principles for scientific data management and stewardship". Scientific Data (2016). 10.1038/sdata.2016.18 PMC4792175 26978244

[36] McLennan S, Shaw D, Celi LA (2018) The challenge of local consent requirements for global critical care databases. 10.1007/s00134-018-5257-y 29922844

[37] Guinchard A. Taking proportionality seriously: The use of contextual integrity for a more informed and transparent analysis in EU data protection law. 23 4 2018 https://onlinelibrary.wiley.com/doi/abs/10.1111/eulj.12273

[38] LeVeque R.J. Top Ten Reasons to Not Share Your Code (and why you should anyway). Retrieved November 1, 2018, from https://faculty.washington.edu/rjl/pubs/topten/topten.pdf

[39] Barnes N. Publish your computer code: it is good enough. Nature 467, 753 (2010). https://www.nature.com/news/2010/101013/full/467753a.html 10.1038/467753a 20944687

[40] Wittek P. PLOS Blogs: Stop hiding your code. Retrieved November 1, 2018, from https://blogs.plos.org/blog/2018/04/18/stop-hiding-your-code/

[41] Eglen SJ, Marwick B, Halchenko YO, Hanke M, Sufi S, Gleeson P, et al Toward standard practices for sharing computer code and programs in neuroscience. Nature Neuroscience. 2017 5 25;20(6):770 10.1038/nn.4550 28542156 PMC6386137

